# The value of combined vein resection in pancreaticoduodenectomy for pancreatic head carcinoma: a meta-analysis

**DOI:** 10.1186/s12893-019-0540-6

**Published:** 2019-07-08

**Authors:** Cheng Peng, Di Zhou, Lingjun Meng, Yanlong Cao, Hanwen Zhang, Zheng Pan, Chao Lin

**Affiliations:** 1 Department of Hepatobiliary-Pancreatic Surgery, China-Japan Union Hospital of Jilin University, 126 Xiantai Street, Changchun, 130033 China; 2 Department of Hematology and Oncology, China-Japan Union Hospital of Jilin University, Changchun, 130033 China; 3 Department of general surgery, Xi’an No.4 Hospital, Xi’an, 710000 China

**Keywords:** Pancreaticoduodenectomy, Vein resection, Meta-analysis, Pancreatic cancer

## Abstract

**Background:**

Although pancreaticoduodenectomy with vein resection (PDVR) is widely performed in selected patients with indications, its benefits remain controversial. In this meta-analysis, we evaluate the safety and efficacy of PDVR in comparison to standard pancreaticoduodenectomy (PD).

**Methods:**

We searched PubMed, Embase, and Cochrane as well as the Chinese National Knowledge Infrastructure, Weipu, and Wanfang databases for studies that evaluate the value of PVDR. The data of the patients who underwent PD or PDVR were analyzed using Review Manager and STATA software.

**Results:**

In comparison with the PD group, the PDVR group had a lower R0 resection rate and higher rates of complications such as biliary fistula, reoperation rate, delayed gastric emptying, cardiopulmonary abnormalities, hemorrhage, in-hospital mortality, 30-day mortality. The blood loss, duration of operation, total hospital stay is higher in PDVR group.

**Conclusions:**

Compared to standard PD, PDVR was associated with a greater risk of some specific complications and increase the mortality rate, total hospital stay time, combine with vein resection have a lower R0 resection rate. Therefore, combine with vascular resection for pancreatic cancer needs to be carefully selected by the surgeon.

## Background

Pancreatic ductal adenocarcinoma (PDAC) accounts for 90% of pancreatic malignant neoplasms and remains the digestive cancer with the poorest prognosis, with a 5-year overall survival rate of 7–8% [1]. Pancreaticoduodenectomy (PD) is the only surgical option for the management of pancreatic head cancer. The major goal of surgery is to achieve R0 resection to be potentially curative [2]. Therefore, to ensure that the post-resection surgical margin of the tumor is negative for cancer cells (R0 resection), the use of PD is restricted to patients who have no borderline resectable lesions or locally unresectable lesions and have no metastatic disease [3, 4]. Another point in consideration is that only 15–20% of patients are candidates for surgical resection, after careful pre-therapeutic evaluation [1]. Further complications arise if the tumor invades major vascular structures adjacent to the pancreatic head, such as the portal vein (PV) and superior mesenteric vein. In some cases, PD combined with vein resection (PDVR) may be performed in an attempt to achieve a negative surgical margin [5, 6]. While PVDR is no longer considered an absolute contraindication in pancreatic head cancer, the benefits of PDVR still remain debatable. In the past, studies have shown that the median overall survival of patients undergoing PVDR for borderline and locally advanced pancreatic cancer is 22 to 24.9 months [7, 8]. On the other hand, some studies have shown that vein resection and reconstruction performed along with PD do not increase the complication rate and postoperative mortality and that the procedure is a safe and feasible option to improve the tumor resection rate [9–11]. In contrast, other studies have evaluated the risk of surgery and the overall survival outcomes and concluded that an operative intervention for patients with pancreatic cancer is not favorable [12, 13]. These results further emphasize the importance of determining whether PDVR actually benefits patients with borderline tumors that are not amenable to R0 resection with conventional PD; improving the R0 resection rate would enhance the cost-effectiveness of the surgery and improve survival and quality of living.

Thus, there is still some ambiguity regarding the benefits of PVDR. In this study, we aimed to conduct a systematic review and meta-analysis of available literature to compare the complications and survival benefits of PDVR and PD (performed with the classical technique or with preservation of the pylorus).

## Methods

### Search strategy

We conducted a systematic search of various international and national databases, including PubMed, EMBASE, the Cochrane Library, Chinese National Knowledge Infrastructure, Wanfang Database, and Weipu database. No restrictions were placed on language or publication year. The following search terms were used as key words for the database search: “pancreaticoduodenectomy,” “duodenopancreatectomy,” “Whipple,” “vascular resection,” “venous resection,” “vein resection,” “portal vein resection,” “superior mesenteric vein resection,” “venous reconstruction,” “venous reconstruction,” and “vascular reconstruction.” The search was performed in January 2019. Moreover, additional potentially eligible studies were obtained by a manual search of the references of relevant reviews.

### Inclusion and exclusion criteria

Relevant clinical trials were selected according to the following inclusion criteria: (1) The patients enrolled were those who underwent PD or PDVR for malignancy of the pancreatic head. (2) The study compared PD and PDVR in terms of surgical procedures, postoperative complication rates, tumor characteristics, duration of hospitalization, or survival rates.

The studies were excluded if they met any of the following conditions: (1) The study included patients with malignancy of the pancreatic body or tail or periampullary tumors (ampullary carcinoma, distal bile duct cholangiocarcinoma, and duodenal carcinomas), with PD, PDVR, total pancreatectomy, distal pancreatectomy, or central resection being performed according to the tumor location. (2) The papers were non-comparative studies, reviews, commentaries, or case reports. (3) Studies did not provide sufficient data. (4) Papers were duplicate publications.

### Data extraction and study quality assessment

One investigator extracted all data from the selected studies, while the other independently re-extracted the data and corrected them. Disagreements were resolved by mutual consensus. Data extracted from eligible articles for analysis included the following: (1) the first author’s name, year of publication, country, and study design; (2) the number of patients in the experimental group and control group as well as their age and male ratio; and (3) data regarding surgical procedures, postoperative complication, tumor characteristics, duration of hospitalization, and survival.

Since the rate of R0 resection will be a major finding in this study, all the included studies containing the data related to R0 resection were assessed thoroughly to determine whether they referred to the AJCC guidelines to define R0 resection. According to the AJCC guidelines. R0 indicates no evidence of residual tumor. R1 indicates presence of microscopic tumor at margins, as defined by College of American Pathologists (CAP); however, the Royal College of Pathologists (RCP) R1 definition includes tumors within a 1-mm margin. Macroscopically visible tumor at margins is classified as R2.

The quality of the included studies was assessed by two independent investigators using the Newcastle–Ottawa Scale [14]. Each article was assigned a score between 0 and 9 for the parameters of patient selection, comparability, and outcome. A score ≥ 7 indicated that the study was of high quality with a low risk of bias.

### Data analysis and synthesis

Data were presented as means and SDs for continuous variables and number of cases for dichotomous variables. All statistical analyses were carried out using RevMan 5.3 to generate the odds ratios (OR), mean difference (MD), and their corresponding 95% confidence intervals (CI). Heterogeneity was assessed using the I^2^ statistic, with I^2^ ≥ 50% indicating substantial heterogeneity. For studies that showed heterogeneity, we sought to determine the possible source of heterogeneity and then used the random effects model for further analysis [15, 16]. If I^2^ was < 50%, the fixed effects model was applied. Publication bias was estimated by visual assessment of funnel plots. All *P* values were two-sided, and a P value of < 0.05 was considered statistically significant.

## Results

### Characteristics of included studies

**Fig. 1:**
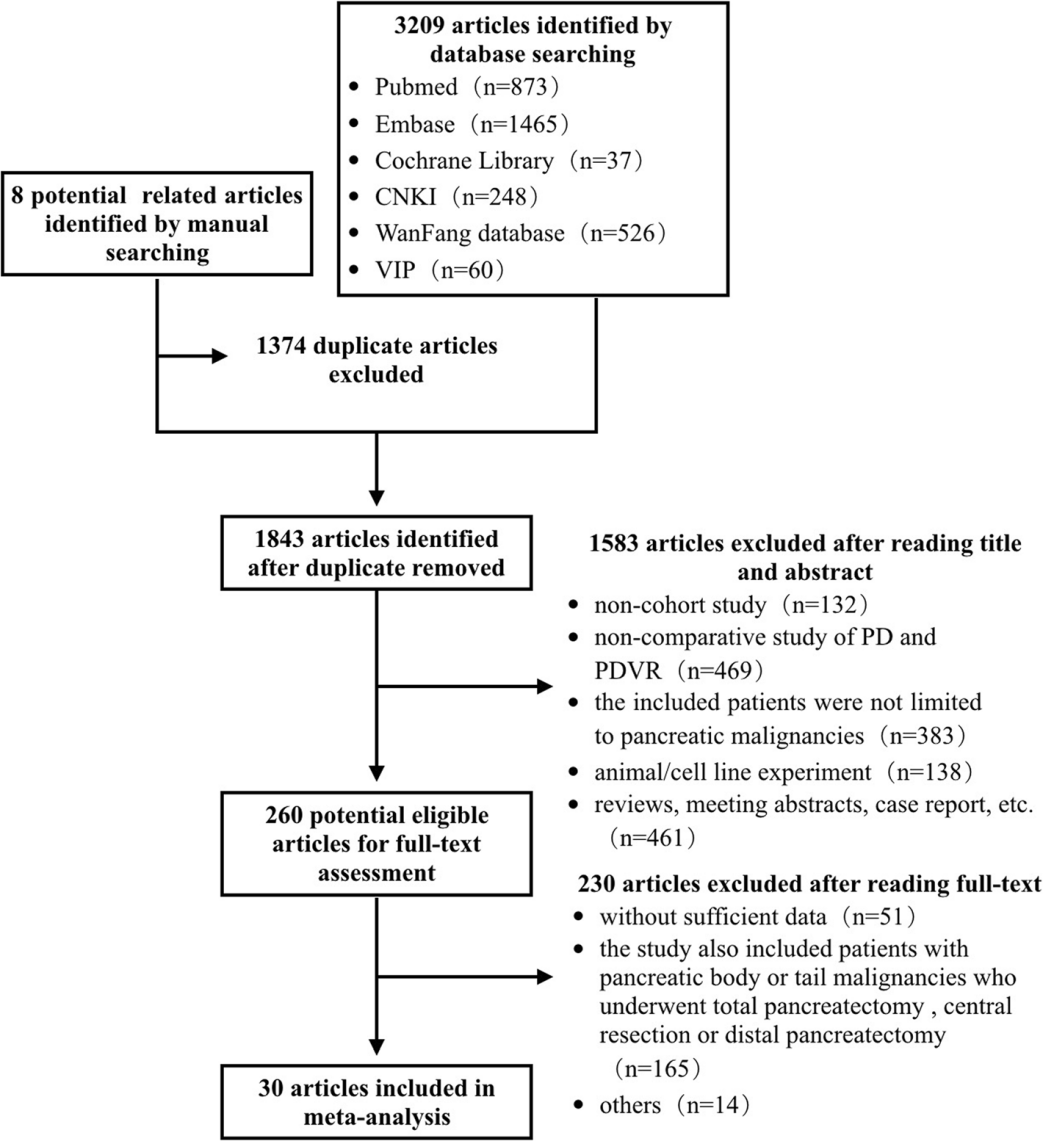
Flow diagram of the studies identified in the meta-analysis

We were able to extract 3217 publications from the online databases using the search terms. In addition, 8 publications were identified by manual searching. After eliminating duplicate records and irrelevant papers by reading titles and abstracts, 260 articles were selected for full-text assessment. Finally, 30 studies comprising 12,031 patients (2186 who underwent PDVR and 9845 who underwent PD) were chosen for the meta-analysis. The study screening process has been summarized in Fig. 1. Briefly, the included studies were published between 1996 and 2017. Of the 30 included studies, 13 were from the USA; 6 were from China; 3 each were from Japan and the UK; 2 were from France; and 1 each was from Korea, Australia, and Turkey [11, 17–45]. All the studies included were retrospective cohort studies and investigated patients who underwent PD or PDVR for malignancy of the pancreatic head.

The results of the quality assessment of the studies, with the Newcastle–Ottawa Scale scores for each study, are summarized in Table 1. Of the 30 studies, 28 were of high quality, with scores between 7 and 9; the remaining 2 studies had scores of 6 points.

**Table 1:** The characteristics and results of the quality assessment of the included studies

First author	Publication year	Country	Study design	PDVR group			PD group			Newcastle-Ottawa Scale			
				No.	Age	Male (%)	No.	Age	Male (%)	selection	comparability	outcome	score
Lawrence E	1996	USA	cohort	42	n.a.	n.a.	231	n.a.	n.a.	****	*	*	6
S. D. LEACH	1998	USA	cohort	31	mean 66.0	19 (61)	44	mean 64.0	23 (52)	****	*	***	8
Tbomas	2003	USA	cohort	13	68.0 ± 13.0	7 (53.8)	23	67.0 ± 8.6	14 (60.9)	****	**	*	7
Ronnie T	2004	China	cohort	12	n.a.	n.a.	38	n.a.	n.a.	****	*	**	7
Jennifer F	2004	USA	cohort	110	64 (41–81)	69 (62.7)	181	64 (30–83)	106 (58.6)	****	**	**	8
Nicolas Carrere	2006	France	cohort	45	58.8 ± 1.7	32 (71.1)	88	61.5 ± 1.1	59 (67.0)	****	**	**	8
Isao Kurosaki	2008	Japan	cohort	35	66.2 ± 9.2	19 (54.3)	42	64.1 ± 8.8	24 (57.2)	****	**	***	9
Robert C	2009	USA	cohort	31	n.a.	n.a.	36	n.a.	n.a.	****		**	6
Paul Toomey	2009	USA	cohort	48	67.0 ± 9.2	27 (56.3)	172	68.0 ± 7.8	80 (46.5)	****	*	***	8
Yuji Kaneoka	2009	Japan	cohort	42	66.0 ± 1.0	24 (57.1)	42	65.0 ± 2.0	28 (66.7)	****	*	***	8
K Dilip Chakravarty	2010	China	cohort	12	62.9 ± 11.0	7 (58.3)	75	62.9 ± 9.8	50 (66.7)	****	**	***	9
VM.Banz	2011	UK	cohort	51	67 (46–80)	24 (47.1)	275	65 (27–83)	147 (53.5)	****	**	*	7
Anthony W. Castleberry	2012	USA	cohort	281	65.5 ± 11.2	138 (49.1)	3301	65.6 ± 11.4	1701 (51.5)	****	*	**	7
Ryan S. Turley	2012	USA	cohort	42	64 (40–78)	22 (62)	162	66 (32–87)	81 (50)	****	*	***	8
Reena Ravikumar	2013	UK	cohort	230	65 (43–80)	115 (50)	840	66 (27–84)	468 (55.7)	****	**	***	9
yoshiaki murakami	2013	Japan	cohort	61	n.a.	33 (54.1)	64	n.a.	32 (50.0)	****	**	**	8
Jaehong Jeong	2013	Korea	cohort	46	61 (41–81)	30 (65.2)	230	62 (32–80)	129 (56.1)	****	**	**	8
Vijay G	2013	USA	cohort	18	mean 67.2	6 (33.3)	43	69	21 (48.8)	****	**	*	7
Ali Aktekin	2013	Turkey	cohort	23	64.73	7 (30.4)	77	63.6 ± 11.8	49 (63.6)	****	*	***	8
Yi Gong	2013	China	cohort	119	59 (30–82)	72 (60.5)	447	59 (37–81)	295 (66.0)	****	**	**	8
Kaitlyn J. Kelly	2013	USA	cohort	70	66.8 ± 9.1	28 (40)	422	65.0 ± 11.3	214 (51)	****	*	***	8
F.wang	2014	Australia	cohort	64	66 (62–73)	34 (53.1)	58	67 (61–75)	30 (51.7)	****	**	**	8
Tan TO Cheung	2014	China	cohort	32	63 (35–86)	20 (62.5)	46	67 (37–82)	25 (54.3)	****	**	**	8
Alexandra M. Roch	2015	USA	cohort	90	66.4 ± 10.4	51 (56.7)	477	66.3 ± 10.4	274 (57.4)	****	**	***	9
H Elberm	2015	UK	cohort	230	n.a.	n.a.	840	n.a.	n.a.	****	*	**	7
Michael D. Sgroi	2015	USA	cohort	60	64.5 ± 10.0	32 (53.3)	87	67.4 ± 9.7	43 (49.4)	****	**	***	9
Wei-lin Wang	2015	China	cohort	42	59.4 ± 8.5	26 (61.9)	166	60.5 ± 12.3	115 (69.3)	****	*	**	7
Xin Zhao	2016	China	cohort	21	63.0 ± 7.5	13 (61.9)	85	63.5 ± 10.7	44 (51.8)	****	*	**	7
Joal D	2016	USA	cohort	194	65.0 ± 11.2	86 (44.3)	1163	64.3 ± 11.8	603 (51.8)	****	*	**	7
Pietro Addeo	2017	France	cohort	91	66.0 ± 10.0	52 (57.1)	90	69.0 ± 9.0	54 (60)	****	**	***	9

*Scores obtained in this domain

### Surgical procedures and hospitalization

**Fig. 2:**
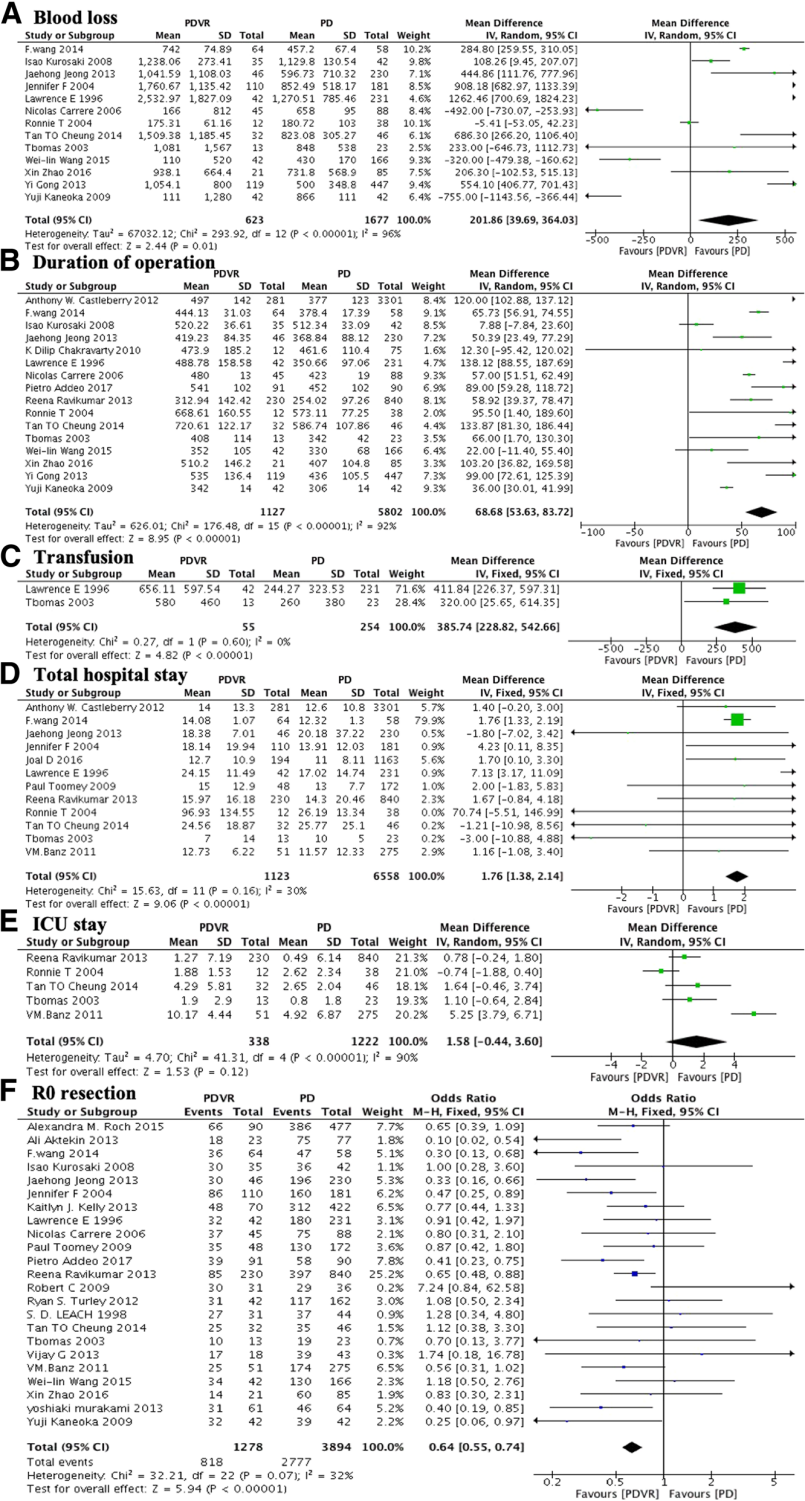
Comparison of PDVR and PD by surgical procedures and hospitalization

In contrast to the PD group, the PDVR group had greater operative blood loss (MD: 201.86; 95% CI: 39.69 to 364.03; *P* = 0.01; I^2^ = 96%; Fig. 2a) and longer operative time (MD: 68.68; 95% CI: 53.63 to 83.72; *P* < 0.001; I^2^ = 92%; Fig. 2b). However, the significant heterogeneity in the studies weakened the power of the conclusion. Further, the PDVR group also had greater volume of intraoperative transfusion (MD: 385.74; 95% CI: 228.82 to 542.66; *P* < 0.001; I^2^ = 0%; Fig. 2c) and longer duration of hospitalization (MD: 1.76; 95% CI: 1.38 to 2.14; P < 0.001; I^2^ = 30%; Fig. 2d). No statistically significant intergroup differences were noted in terms of the length of intensive care unit (ICU) stay (MD 1.58; 95% CI: − 0.44 to 3.60, *P* = 0.12, I^2^ = 90%, Fig. 2e). Further, 23 out of the 30 included studies contain the data related to R0 resection, all these studies referred to the AJCC guidelines to define R0 resection. The PDVR group had a lower rate of R0 resection than the PD group (64.0% versus 71.3%; OR 0.64; 95% CI: 0.55 to 0.74; *P* < 0.001; I^2^ = 32%; Fig. 2f).

### Mortality

**Fig. 3:**
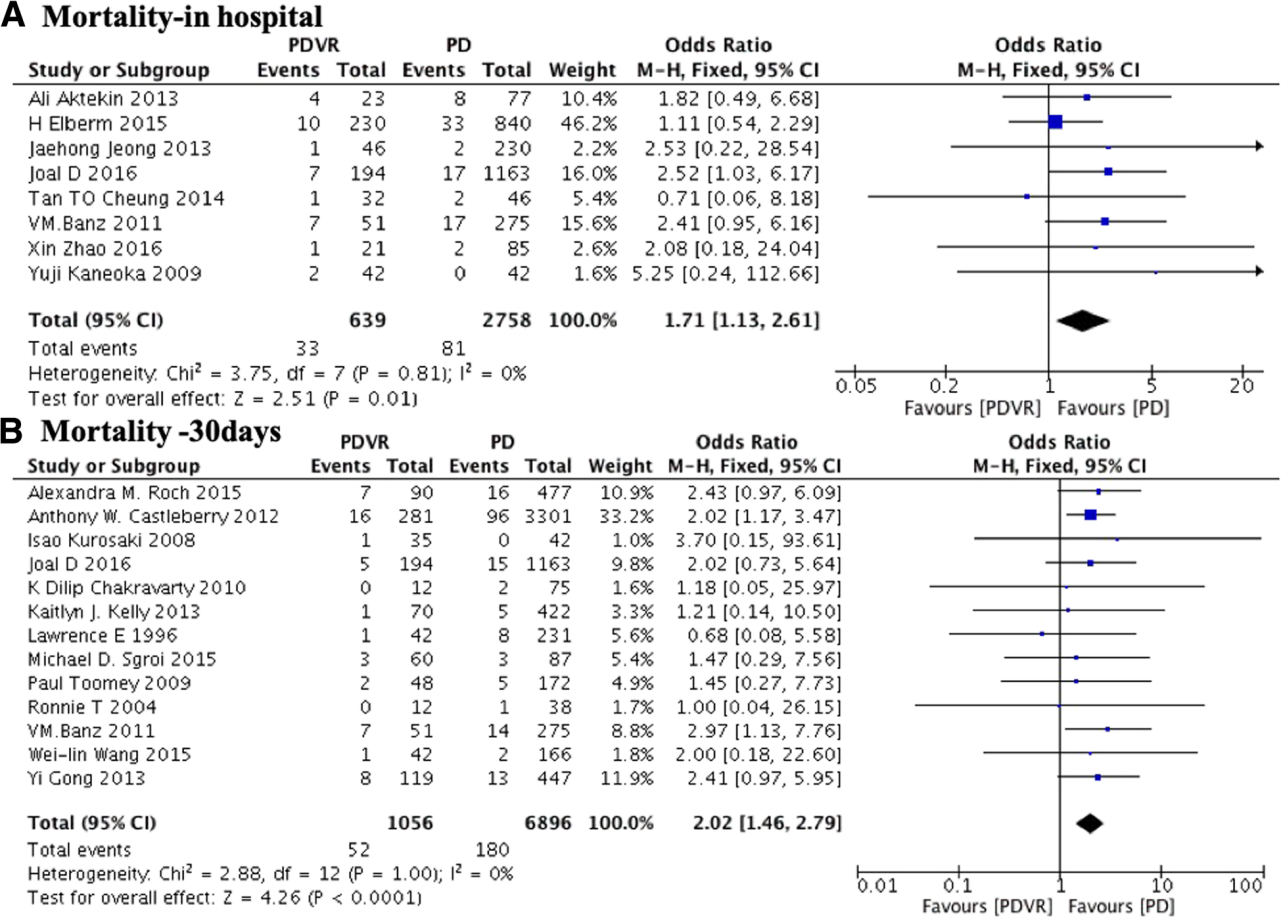
Comparison of PDVR and PD by mortality

The rate of in-hospital mortality (5.2% versus 2.9%; OR: 1.71; 95% CI: 1.13 to 2.61; *P* = 0.01; I^2^ = 0%; Fig. 3a) as well as 30-day mortality (4.9% versus 2.6%; OR 2.02; 95% CI: 1.46 to 2.79; P < 0.001, I^2^ = 0%, Fig. 3b) were higher in the case of the PDVR group as compared to the PD group.

### Oncological outcome

**Fig. 4:**
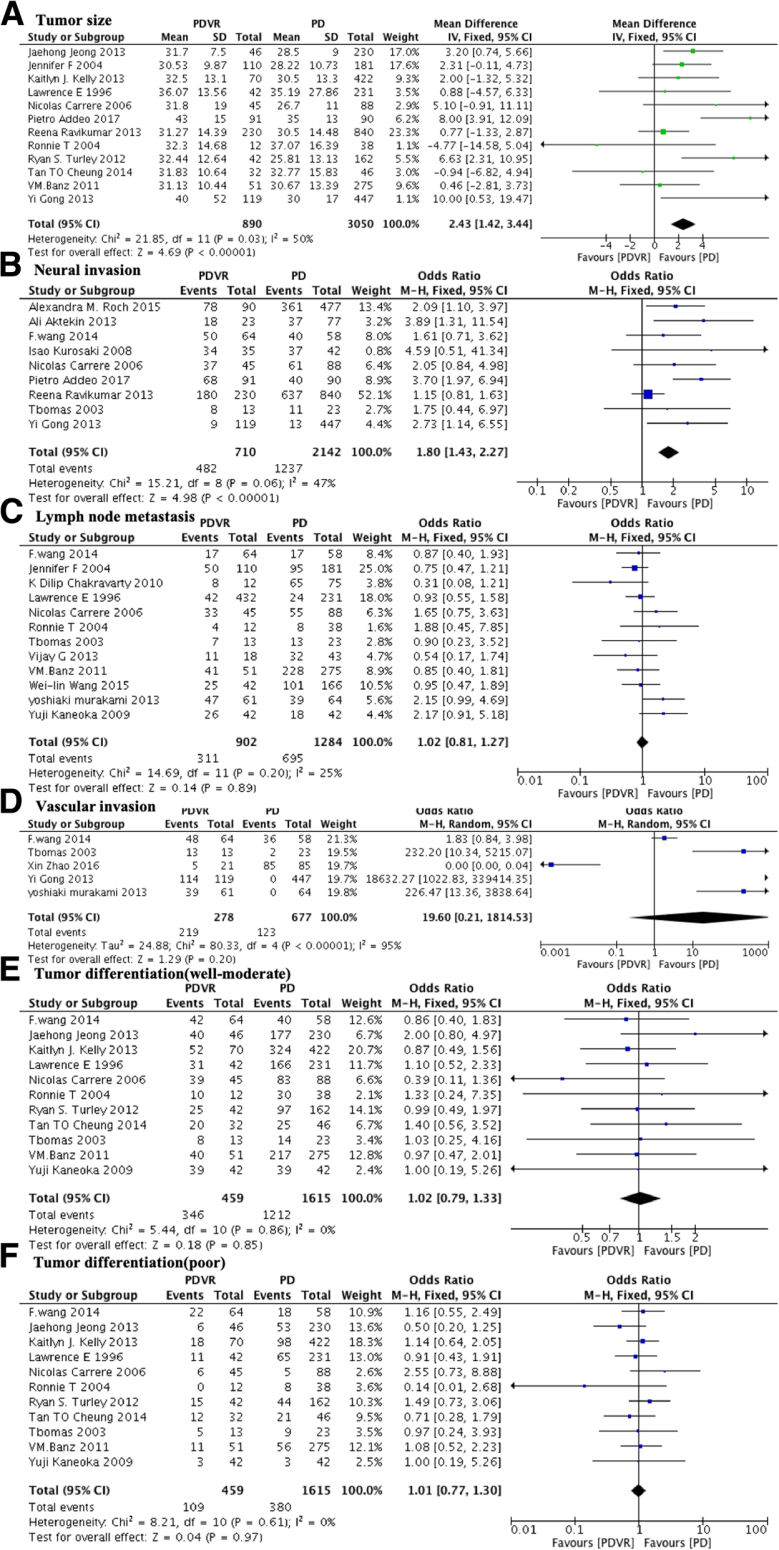
Comparison of PDVR and PD by oncological outcome

Compared to the PD group, the PDVR group had a greater tumor size (MD 2.43; 95% CI: 1.42 to 3.44; P < 0.001; I^2^ = 50%; Fig. 4a) and a higher neural invasion rate (67.9% versus 57.7%; OR: 1.82; 95% CI: 1.43 to 2.27; P < 0.001; I^2^ = 47%; Fig. 4b). However, no significant differences between the PD and PDVR groups were noted for the following tumor parameters: lymph node metastasis rate (34.5% versus 54.1%; OR 1.02; 95% CI: 0.81 to 1.27; *P* = 0.89; I^2^ = 25%; Fig. 4c), vascular invasion rate (78.8% versus 18.2%; OR: 19.60; 95% CI: 0.21 to 1814.53; *P* = 0.02; I^2^ = 95%; Fig. 4d), well to moderate tumor differentiation rate (75.4% versus 75.0%; OR: 1.02; 95% CI 0.79 to 1.33; *P* = 0.85; I^2^ = 0%; Fig. 4e), or poor tumor differentiation rate (23.7% versus 23.7%; OR: 1.01; 95% CI: 0.77 to 1.30; *P* = 0.97; I^2^ = 0%; Fig. 4f).

### Postoperative complications

In the present meta-analysis, 12 out of the 30 included studies contain data related to the occurrence rate of pancreatic fistula, 11 of them defined pancreatic fistula as a drain output of any measurable volume of fluid on or after postoperative day 3 with an amylase content greater than 3 times the serum amylase activity, which was published by International Study Group on Pancreatic Fistula Definition in 2005 [46]. Only 1 study published in 2003 defined pancreatic fistula as drainage of more than 50 ml of fluid with an amylase concentration greater than three times the upper limit of normal serum level after postoperative day 10.

**Fig. 5:**
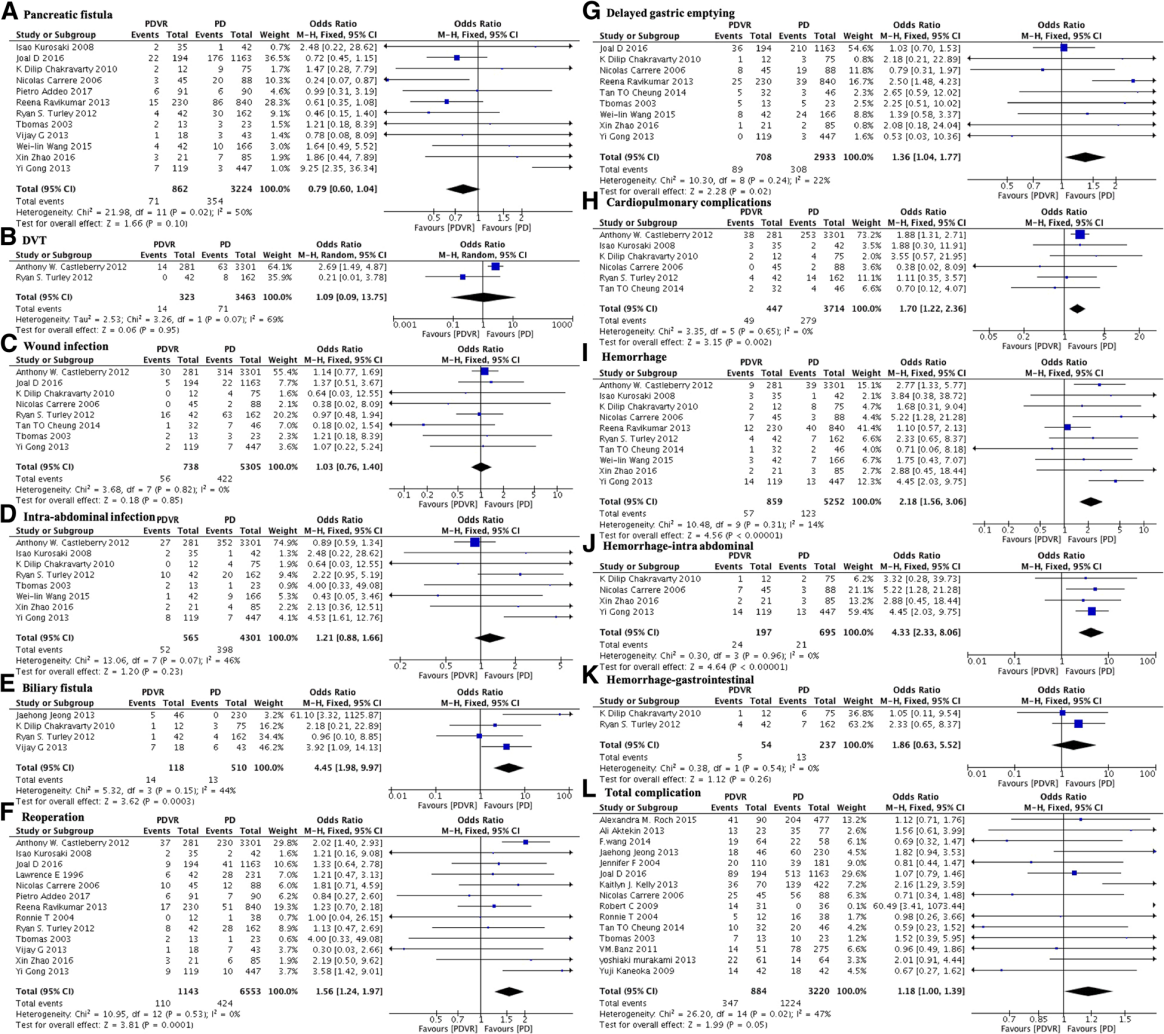
Comparison of PDVR and PD by postoperative complications

With respect to postoperative complications, the current meta-analysis revealed that both the PDVR and PD groups had a similar incidence of pancreatic fistula (8.2% versus 11.0%; OR 0.79; 95% CI 0.60 to 1.04; *P* = 0.10; I^2^ = 50%; Fig. 5a), incidence of deep vein thrombosis (4.3% versus 2.1%; OR 1.09; 95% CI 0.09 to 13.75; *P* = 0.95; I^2^ = 69%; Fig. 5b), wound infection rate (7.6% versus 8.0%; OR 1.03; 95% CI 0.76 to 1.40; *P* = 0.85; I^2^ = 0%; Fig. 5c), and intra-abdominal infection rate (9.2% versus 9.3%; OR 1.21; 95% CI 0.88 to 1.66; *P* = 0.23; I^2^ = 46%; Fig. 5d).

Compared to the PD group, the PDVR group showed higher rates of complications such as biliary fistula (11.9% versus 2.5%; OR: 4.45; 95% CI: 1.98 to 9.97; *P* < 0.001; I^2^ = 44%; Fig. 5e), reoperation rate (9.6% versus 6.5%; OR 1.56; 95% CI: 1.24 to 1.97; P < 0.001; I^2^ = 0%, Fig. 5f), delayed gastric emptying (12.6% versus 10.5%, OR 1.36, 95% CI: 1.04 to 1.77, *P* = 0.02, I^2^ = 22%, Fig. 5g), cardiopulmonary abnormalities (11.0% versus 7.5%; OR 1.70; 95% CI: 1.22 to 2.36; *P* = 0.002; I^2^ = 0%; Fig. 5h), and hemorrhage (6.6% versus 2.3%, OR: 2.18, 95% CI: 1.56 to 3.06; P < 0.001; I^2^ = 14%; Fig. 5i). In addition, some studies analyzing the rates of postoperative hemorrhage occurring at different sites showed that the rate of intra-abdominal hemorrhage (12.2% versus 3.0%; OR: 4.33; 95% CI: 2.33 to 8.06; P < 0.001; I^2^ = 0%; Fig. 5j) was greater in the PDVR group, while the rate of gastrointestinal hemorrhage (9.3% versus 5.5%; OR: 1.86; 95% CI: 0.63 to 5.52; *P* = 0.26; I^2^ = 0%; Fig. 5k) was similar in both groups.

Fifteen of the 30 studies provided summarizations of the number of patients with different complications as the total complication rate; with respect to this parameter, the two groups did not show any statistically significant differences (39.3% versus 38.0%; OR 1.18; 95% CI: 1.00 to 1.39; *P* = 0.05; I^2^ = 47%; Fig. 5l).

### Sensitivity analysis and publication bias

**Fig. 6:**
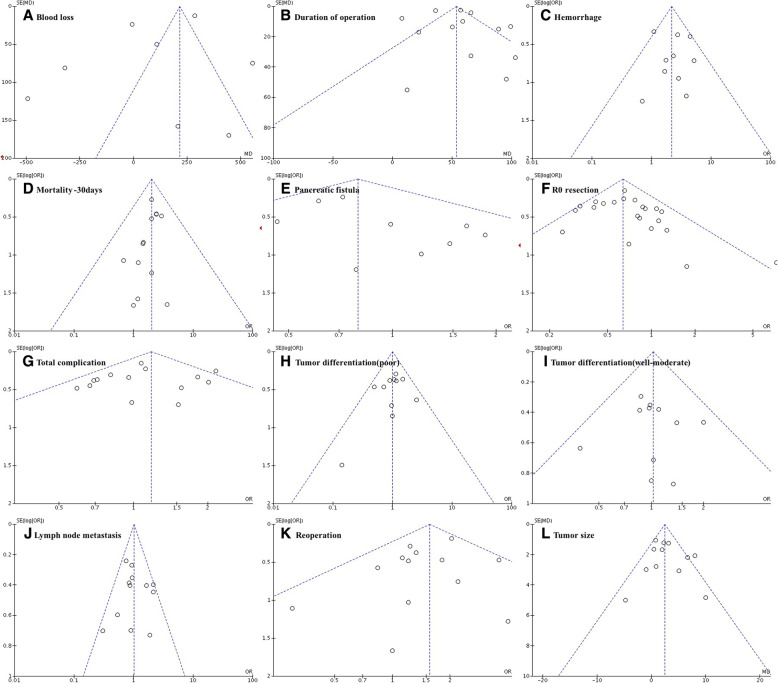
Funnel plots for publication bias assessment

We further sought to examine the influence of individual studies on the results of our meta-analysis. We found that the removal of any of the included studies did not have any significant effect on the overall outcome. Most of the reported results had overlapping confidence intervals, which further ensured that our findings were not significantly influenced by any individual study. We checked for the existence of publication bias by preparing funnel plots for comparisons with more than 10 studies. No substantial asymmetry was found by visual inspection of the funnel plots (Fig. 6).

## Discussion

PDVR is important in clinical practice and is technically complex and requires considerable surgical skills. Invasive pancreatic cancer can easily progress, with infiltration of the adjacent nerves and important vascular structures such as the superior mesenteric vein and portal vein. Therefore, the question of whether vascular resection should be combined with surgical resection of pancreatic cancer is an important concern.

A few meta-analyses on PDVR have been reported in the past [47–50]. Song and colleagues focused on the effect of different interposition grafts in PDVR [49]. In contrast with the meta-analyses performed by F. Giovinazzo [48], Richard Bell [47] and Yu [50], our meta-analysis included the greatest amount of studies with a relatively high quality; thus, our results are more representative. In addition, we completed a comprehensive analysis for the purpose of presenting the most complete data, including surgical procedures, hospitalization, mortality, oncological outcome, and postoperative complications. Some of the parameters were assessed have not been included in previous meta-analyses, but data regarding these parameters may facilitate the decision-making process for clinicians. In terms of R0 resection rate, which is the major finding of our study, our results were consistent with those of Giovinazzo et al. [48] and Bell et al. [47]. Another difference is that we included only data pertaining to patients who had malignancy of the pancreatic head for which they underwent pancreaticoduodenectomy, which ensured the homogeneity of the research population and reduced bias. Previous analyses have shown poor survival outcomes after PDVR and do not recommend this aggressive surgical approach [51, 52]. However, PDVR continues to be performed for pancreatic cancer at some centers [27, 33, 53].

Surgeons continue to debate on whether combined vascular resection can increase the R0 resection rate of pancreatic head cancer. Analyses of the pathology outcomes showed that the PDVR group had a greater tumor size, higher neural invasion rates, and lower R0 resection rates than the PD group. However, there were no differences between the two groups in terms of lymph node metastasis, vascular invasion, or type of tumor differentiation (poor or well–moderate). Taken together, these findings imply that patients in the PDVR group have a higher probability of local infiltration of disease without an increased frequency of lymph node metastasis. In addition, the type of tumor differentiation was similar for both groups.

With respect to postoperative complications, the PDVR group showed a greater rate of the biliary fistula, reoperation, delayed gastric emptying, cardiopulmonary abnormalities, hemorrhage, as well as a longer duration of hospitalization. Other investigators have also indicated that patients undergoing vascular resection have a higher rate of complications [38, 54]. The longer duration of hospitalization may be attributed to these complications. However, the two groups in our study did not show any differences in the rate of complications such as pancreatic fistula, deep vein thrombosis, wound infection, ICU stay, or total rate of complications. The incidence rate of biliary fistula in the PDVR group increased significantly, but not the pancreatic fistula. We speculated that it is the ischemic necrosis of the bile duct that lead to the difference. During the process of vascular resection and reconstruction, the PDVR group has a greater chance of blood vessels damage in the hepatoduodenal ligament than PD group, especially some tiny blood vessels and collateral circulation. This may indirectly decrease the blood supply of the residual bile duct, which may lead to bile duct ischemic necrosis and biliary fistula. Compared with bile duct, pancreas has a more sufficient blood supply and collateral circulation thanks to its innate anatomical characteristics. Therefore, the effect of PDVR on blood supply of pancreas is not as great as that of bile duct.

Patients who undergo PD also develop various complications, which may even be fatal. Combined vein resection increases the occurrence of postoperative complications in patients, thereby increasing the risks for patients undergoing PVDR during the postoperative period. However, the total rate of complications, which is defined as the proportion of patients with any kind of complication to the total patient population, did not differ between the two groups. Nevertheless, it is worth mentioning that a patient can have multiple complications. Therefore, although there are significant differences between PD group and PDVR group in some specific complications, the total complication rate does not necessarily differ significantly between the two groups.

The mortality associated with combined vascular resection is also a valuable point of consideration. In this meta-analysis, we observed that the 30-day and in-hospital mortality rates were indeed greater in the PDVR group. Although there were no significant differences in the total number of complications between the two groups, the rates of cardiopulmonary complication, hemorrhage, and reoperation were higher in the PDVR group than the PD group. Could the increase in mortality during these 2 periods be attributed to any of the abovementioned complications? As mentioned above, it is possible for a postoperative patient to have multiple complications at the same time, so once complications occur, the patient is often in a very serious condition and has a high mortality rate. Further investigation focusing on this question would be necessary to arrive at suitable answers.

According to NCCN guidelines [55], neoadjuvant therapy, including chemotherapy and chemoRT, has the potential to downsize tumors to increase the likelihood of a margin-free resection. It can be considered after biopsy confirmation. Most NCCN Member Institutions now prefer an initial approach for patients with borderline resectable disease that involves neoadjuvant therapy, as opposed to immediate surgery; upfront resection in patients with borderline resectable disease is no longer recommended. Neoadjuvant therapy is also sometimes used in patients with resectable disease, especially in those with high-risk features. Several trials have demonstrated that for patients with borderline resectable lesions, neoadjuvant therapy can be effective and well-tolerated [56–58]. In the present meta-analysis, 25 out of the 30 included studies did not present the information regarding neoadjuvant therapy; only 5 studies mentioned that some patients in PD group and PDVR group received neoadjuvant therapy, but they did not conduct a separate comparison between these patients who received neoadjuvant therapy. Regrettably, in our meta-analysis, we not able to independently analyze these patients.

Our study has a few limitations. In the pooled analysis of blood loss, duration of operation, ICU stay, vascular invasion, and DVT, the heterogeneity is relatively high. We have tried to determine the possible source of heterogeneity from the perspective of professional knowledge, but we failed to find the sources of heterogeneity. Therefore, we applied the random effect model, which may affect the credibility of the results to some extent. Another limitation is the lack of uniformity among the various studies with respect to the defining criteria for the various complications; this could lead to deviations in the data collected. Nevertheless, our study does have some merits. We employed a comprehensive search strategy and applied clearly defined, strict inclusion and exclusion criteria. The most recent studies were included, and 90% of these studies were of high quality, with minimal heterogeneity of results among the studies.

## Conclusions

Compared to standard PD, PDVR appears to be associated with a greater risk of some specific complications and increase the mortality rate, total hospital stay time. Combine with vein resection have a lower R0 resection rate. On the basis of the results of this meta-analysis, we recommend that combine with vascular resection for pancreatic cancer needs to be carefully selected by the surgeon.

## Data Availability

Please contact author for data requests.

## Abbreviations

CAP: College of American Pathologists
CI: Confidence intervals
ICU: Intensive care unit
MD: Mean difference
OR: Odds ratios
PD: Pancreaticoduodenectomy
PDAC: Pancreatic ductal adenocarcinoma
PDVR: Pancreaticoduodenectomy with vein resection
PV: Portal vein
RCP: Royal College of Pathologists
